# Initiation of Paliperidone palmitate 3-monthly injectable in an acute inpatient psychiatric unit

**DOI:** 10.1192/j.eurpsy.2024.1512

**Published:** 2024-08-27

**Authors:** A. S. Pareja Zea, C. Vera-Varela, A. Martínez Martínez, G. García Jimenez, M. De Iceta Ibañez de Gauna

**Affiliations:** ^1^Psychiatry; ^2^Pharmacy, Infanta Sofia University Hospital, Madrid, Spain

## Abstract

**Introduction:**

The treatment of patients with severe psychotic disorders presents significant clinical challenges, and the choice of appropriate therapy is essential to ensure long-term stability^1^. In this context, long-acting injectable antipsychotics (LAIs) have emerged as a promising therapeutic option. LAIs were developed to counteract poor treatment adherence in patients with psychotic disorders^2^.

Paliperidone palmitate 3-monthly injectable (PP3M) is a novel formulation of intramuscular injectable paliperidone palmitate with a significantly longer half-life than the once-monthly formulation.

PP3M has shown a longer time to relapse and good safety and tolerability in many studies^3^.

**Objectives:**

The aim of this work was to describe the profile of patients initiating PP3M in an acute inpatient psychiatric unit.

**Methods:**

A descriptive study was conducted on patients admitted to the acute psychiatric unit from January 2021 to December 2022. The sample included 23 inpatients who initiated PP3M during the admission. Data were collected regarding age, gender, diagnosis, substance abuse, previous antipsychotic treatment, antipsychotic treatment adherence and adverse effects during the admission.

**Results:**

23 patients sample, with an average age of 44.04 years-old, 16 male and 7 female, diagnosed with psychotic disorder (22) and schizoaffective disorder (1). Out of the 23 patients, 7 had active substance abuse upon admission.

Out of the total simple, 9 of them were prescribed LAIs, with 6 on PP1M (Paliperidone palmitate 1-monthly injectable), 2 on PP3M, and 1 on aripiprazole long-acting injection. Twelve were prescribed oral antipsychotics, including 4 on paliperidone, 4 on risperidone, 1 on aripiprazole, 1 on olanzapine, and 2 on other oral antipsychotics. Two patients did not have a previous antipsychotic prescription.

Among the 23 patients, 17 of them did not have previous antipsychotic treatment adherence.

5 out of the 23 patients experienced adverse effects, with 3 of them having extrapyramidal symptoms and 2 hyperprolactinemia.Upon discharge, 11 out of the 23 patients were prescribed antipsychotic monotherapy with PP3M.

**Conclusions:**

In this sample, we observed that inpatients who initiated PP3M in an acute psychiatric unit were males, with psychotic disorders, lacked adherence to previous antipsychotic treatment. Most of them did not experience adverse effects with PP3M during admission.

More research should be done to assess the use of PP3M in an acute inpatient psychiatric unit.

REFERENCES

1. Morris MT, Tarpada SP. Psychopharmacol Bull. 2017 May 15;47(2):42-52.

2. Brasso, C., Bellino, S., Bozzatello, P., Montemagni, C., & Rocca, P. (2017). Neuropsychiatric Disease and Treatment,

3. Edinoff AN, Doppalapudi PK, Orellana C, Ochoa C, Patti S, Ghaffar Y, Cornett EM, Kaye AJ, Viswanath O, Urits I, Kaye AM, Kaye AD. Front Psychiatry. 2021.

**Disclosure of Interest:**

None Declared

